# Length of Stay Comparison between Rivaroxaban and Warfarin in the Treatment of Pulmonary Embolism: Results from a Real-World Observational Cohort Study

**DOI:** 10.1155/2015/414523

**Published:** 2015-12-31

**Authors:** Kirsten M. Roberts, Tamara B. Knight, Eimeira Padilla-Tolentino, Manasa Murthy, Evan J. Peterson

**Affiliations:** ^1^Northwestern Memorial Hospital, 251 E. Huron Street, Chicago, IL 60611, USA; ^2^Seton Northwest Hospital, 11113 Research Boulevard, Austin, TX 78759, USA; ^3^University Medical Center Brackenridge, 601 East 15th Street, Austin, TX 78701, USA; ^4^Seton Medical Center Williamson, 201 Seton Parkway, Round Rock, TX 78665, USA; ^5^Seton Medical Center Austin, 1201 West 38th Street, Austin, TX 78705, USA

## Abstract

*Background*. Trials have shown that novel oral anticoagulants may decrease length of stay versus warfarin. A comparison of length of stay in the treatment of pulmonary embolism (PE) has not been performed outside post hoc analysis of a large clinical trial.* Objective*. To evaluate if rivaroxaban decreases length of stay compared to warfarin plus enoxaparin in the treatment of PE.* Methods*. This was a multicenter, retrospective, observational cohort study. Patients were identified based on discharge diagnosis of PE and were excluded if they received anticoagulants prior to admission and had additional indications for anticoagulation or reduced creatinine clearance. The primary endpoint was length of stay. Secondary endpoints included time from initial dose of oral anticoagulant to discharge and length of stay comparison between subgroups.* Results*. Inclusion criterion was met by 158 patients (82 warfarin, 76 rivaroxaban). The median length of stay was 4.5 days (interquartile range [IQR], 2.7, 5.9) in the warfarin group and 1.8 days (IQR, 1.2, 3.7) in the rivaroxaban group (*P* < 0.001). Time interval from first dose of oral anticoagulant to discharge was shorter with rivaroxaban (*P* < 0.001).* Conclusions*. Patients given rivaroxaban had decreased length of stay versus those given warfarin plus enoxaparin for the treatment of PE.

## 1. Introduction

Pulmonary embolism (PE) is associated with significant morbidity and mortality with sudden death occurring in up to 25% of cases and a cumulative mortality of approximately 30% [1, 2]. Evidence-based guidelines recommend anticoagulation for at least three months following a thromboembolic event [3]. Patients who are not anticoagulated have a risk of recurrence as high as 25% within 14 days of initial diagnosis [4].

Until recently, the standard of care for the treatment of PE was anticoagulation with a vitamin K antagonist (e.g., warfarin) bridged with a parenteral anticoagulant, such as low-molecular weight heparin (LMWH). Within the last four years, four novel oral anticoagulants (NOACs) have been approved by the Food and Drug Administration (FDA) for the treatment of PE. The EINSTEIN [5], RECOVER [6], AMPLIFY [7], and Hokusai-VTE [8] trials found rivaroxaban, dabigatran, apixaban, and edoxaban to be noninferior to warfarin bridged with a parenteral anticoagulant in the treatment of venous thromboembolism (VTE). NOACs offer the advantage of a faster time to reach therapeutic anticoagulation, which is attained after the first dose. In contrast, traditional vitamin K antagonists (VKA) require at least five days of bridging with a parenteral anticoagulant and an INR (international normalized ratio) greater than 2 before desired anticoagulation is achieved.

As the third leading cause of cardiovascular-associated death, VTE imposes a large burden on healthcare systems and resource utilization [9]. In 2011, the total cost of VTE in the United States was $13.5 to $27.7 billion [10]. The reported range of healthcare costs one year following a VTE is $7,594 to $27,909 per patient depending on the clinical scenario [11]. Hospital length of stay (LOS) is one potential contributor to the financial impact of PE [11].

NOACs have been shown to decrease LOS in the setting of both established nonvalvular atrial fibrillation and new-onset nonvalvular atrial fibrillation [12–14]. Laliberté and colleagues found a significant reduction of 0.81 days in those treated with rivaroxaban versus warfarin in patients with nonvalvular atrial fibrillation [12]. Additionally, post hoc analysis of the EINSTEIN trial showed a statistically significant one-day reduction in LOS between patients receiving rivaroxaban versus VKA for the treatment of PE, 6 versus 7 days [13]. LOS comparison between NOACs and VKA with LMWH for the treatment of PE has not been studied in a real-world setting outside of a clinical trial [13, 14].

The objective of this study is to evaluate whether rivaroxaban decreases LOS as compared to warfarin plus enoxaparin for the treatment of PE in the clinical practice setting of a large hospital network.

## 2. Methods

### 2.1. Design

This study was a multicenter, retrospective, observational cohort of patients admitted within a large hospital network consisting of both large teaching hospitals and community hospitals. Patients were identified based on primary discharge diagnosis of PE. Additional data were also accessed by analyzing reports for rivaroxaban medication order. Electronic health records were reviewed for demographic, hospitalization, and discharge information.

### 2.2. Study Population

The study population consists of patients who were discharged between January 1, 2012, and March 1, 2015, with a primary diagnosis of PE based on ICD-9-CM codes (415.1). Patients were excluded if they were receiving anticoagulants prior to admission, had an indication for anticoagulation in addition to pulmonary embolism (e.g., atrial fibrillation), possessed a documented history of coagulopathy in electronic medical records, or were bleeding at the time of admission. Additionally, patients who were pregnant or lactating were excluded. Because rivaroxaban is contraindicated in those with severe renal impairment, patients who had a creatinine clearance of less than 30 mL/minute at the time of admission were excluded. In accordance with trials studying rivaroxaban for the treatment of PE, patients with thrombectomy performed, vena cava filter placement, or a fibrinolytic agent administered were excluded.

### 2.3. Outcomes

The primary endpoint was length of stay. This was defined as the interval from time of admission to time of discharge calculated in days. Secondary endpoints included the interval time from initial dose of rivaroxaban or warfarin to discharge and subgroup analyses. Data regarding baseline characteristics such as comorbid conditions, demographics, and hospital characteristics were also evaluated.

### 2.4. Statistical Analysis

Statistical analyses were performed using SPSS Software Version 17.0 and STATA Version 12.1. Chi-square analysis and Student's *t*-test were used to compare baseline characteristics. Mann-Whitney *U* test and Kolmogorov-Smirnov test were performed for all outcomes measured. Calculations conducted a priori estimated 136 patients would be needed in each arm to detect a 20% relative reduction in the primary outcome, with 80% power at a two-sided significance level of 0.05, assuming an average length of stay of 5.6 days in the warfarin group and 4.5 days in the rivaroxaban group [14, 15]. Subgroup analyses were performed to control for confounding variables. Pearson correlation was performed with length of stay and time from first dose of anticoagulant to discharge. Kaplan-Meier survival analysis was completed to assess percentage of patients hospitalized over time.

## 3. Results

**Figure 1:**
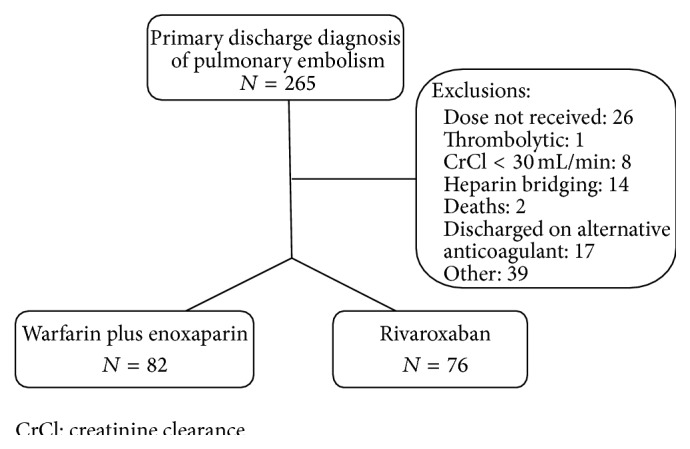
Study flow diagram.

The study included a total of 158 patients. Eighty-two patients met inclusion criteria for the warfarin plus enoxaparin group and seventy-six patients met inclusion for the rivaroxaban group (Figure 1). Baseline characteristics were evenly matched in the groups with the exception of age, diabetes, and coronary artery disease (Table 1). The mean age was 63 ± 15 years in the warfarin plus enoxaparin group and 55 ± 15 years in the rivaroxaban group. Additionally, payer type differed between the groups. Patients in the warfarin plus enoxaparin group were more likely to have Medicare/Medicaid (*N* = 40) or no insurance (*N* = 17) compared to the rivaroxaban group (Medicare/Medicaid *N* = 19; uninsured *N* = 3). There were no differences between the groups with regard to creatinine clearance or hemoglobin upon admission, previous VTE, or PE classification. Most patients presented with stable PE (*N* = 122), while few patients were classified as massive (*N* = 4). The mean INR upon discharge in the warfarin plus enoxaparin group was 1.9 ± 0.9.

**Figure 2:**
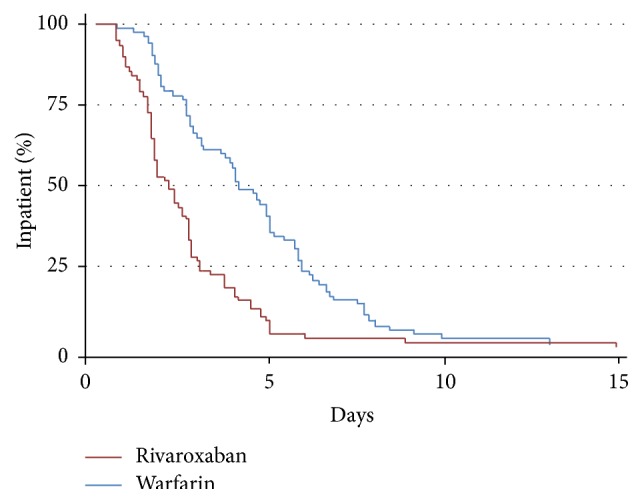
Kaplan-Meier analysis of percentage of patients remaining hospitalized over time in the warfarin plus enoxaparin versus rivaroxaban groups.

For the primary outcome, the median LOS was 4.5 (IQR, 2.7, 5.9) days in the warfarin plus enoxaparin group and 1.8 (IQR, 1.2, 3.7) days in the rivaroxaban group (*P* < 0.001) (Table 2). The time from initial dose of anticoagulant to time of discharge was also significant; the median interval in the warfarin plus enoxaparin group was 3.9 days while the median interval was 0.9 days in the rivaroxaban group (*P* < 0.001) (Table 3). A Kaplan-Meier analysis was performed to illustrate a lower percentage of hospitalized patients over time in the rivaroxaban group compared to the warfarin plus enoxaparin group (Figure 2).

Subgroup analyses were performed according to age, PE classification, and insurance type. A comparison between subjects less than 60 years (*N* = 74) and those greater than or equal to 60 years (*N* = 84) determined that LOS was significantly longer in the warfarin plus enoxaparin group, regardless of age category (Table 2). Additionally, among patients with private insurance LOS was significantly longer with warfarin plus enoxaparin (*N* = 25) versus rivaroxaban (*N* = 54) (*P* < 0.01), but not in patients with Medicare or Medicaid (*P* = 0.09). LOS was also not significantly different in those presenting with a submassive PE between warfarin plus enoxaparin (*N* = 61) and rivaroxaban (*N* = 60) (*P* = 0.06). However, the interval time between initial dose of oral anticoagulant and discharge was significantly shorter in the rivaroxaban group in all subgroups, regardless of payer status or severity classification. Subgroup analyses were not performed on uninsured patients or patients presenting with a massive PE as there were insufficient numbers of patients in one or both treatment groups.

## 4. Discussion

This study is the first to demonstrate a significant reduction in LOS with use of rivaroxaban in the treatment of PE in a real-world setting. A median LOS difference of 2.7 days was found between those who received warfarin plus enoxaparin and rivaroxaban, representing a 60% difference in LOS between the anticoagulants. This is a larger difference than anticipated during initial power analysis and sample size calculations. These results confirm post hoc analysis from the EINSTEIN-PE trial which also found decreased LOS with rivaroxaban use [13, 14]. Upon subgroup analysis, rivaroxaban use in patients with Medicare and Medicaid or being uninsured was not associated with decreased LOS. However, LOS was numerically lower in the rivaroxaban group and this subgroup was underpowered to be able to detect a difference. Similarly, no difference was found in those presenting with a submassive PE. Those with submassive PE had longer LOS regardless of oral anticoagulant received. Despite these findings, rivaroxaban patients were discharged faster after the initial dose of oral anticoagulant in all subgroups, suggesting that the lack of difference in LOS is due to insufficient subject numbers in these subgroups.

This study suggests that older patients tended to receive rivaroxaban less frequently, although this did not appear to affect the primary outcome of LOS. Additionally, patients who have Medicare and Medicaid and/or are uninsured are less likely to receive rivaroxaban. This may be due to practical concerns regarding the higher cost of rivaroxaban compared to warfarin in the outpatient setting.

The authors discuss several reasons for the observed findings. Although most warfarin patients were discharged with subtherapeutic INRs, one possible explanation is that clinicians wanted to observe an appropriate rise in INR prior to discharge. The increased LOS in the warfarin group may also have resulted from the additional time taken to ensure outpatient INR monitoring. Additionally, the high cost of enoxaparin in the outpatient setting may have prevented warfarin patients from being discharged until bridging was complete.

Our results also suggest potential for inpatient cost savings with rivaroxaban based on reduced LOS and medication expenditure. A recent publication by Dasta and colleagues found that the mean total cost of hospitalization for PE was $9,407 and a mean daily cost is approximately $1,735 [16]. Researchers performed further analyses to estimate progression of daily costs. Subsequently, a conservative two-day reduction in LOS would result in estimated $2,985 in cost savings per patient. Purchasing data also supports this conclusion as the estimated inpatient daily cost of rivaroxaban is less than warfarin plus enoxaparin, $14.80 in comparison to $25.28. Therefore, rivaroxaban likely reduces cost of hospitalization by decreased LOS and comparatively lower purchasing prices. Recent studies in the outpatient setting also suggest favorable cost reduction with rivaroxaban use compared to warfarin for commercial healthcare plans [17].

This study has limitations, including its retrospective design. As with any retrospective study, there is the possibility of unmeasured confounding factors affecting the results. There were differences observed in the baseline characteristics between patients in the two treatment arms. We examined subgroups of age category, PE classification, and payer type to see if any of these factors might impact the results as these were factors that were different or could affect LOS. Although mean age was older with warfarin plus enoxaparin, this did not appear to explain the longer LOS because rivaroxaban users had a lower LOS regardless of age category. Payer type may have also affected the results as this could affect both outpatient medication access and posthospitalization support and access to care. Compared to patients with Medicare or Medicaid, patients with private insurance may have had more resources such as home health or easier access for follow-up appointments or INR checks, factors that could expedite discharge. Overall LOS was higher in patients with Medicare or Medicaid, but time to discharge after first dose of oral anticoagulant was lower with rivaroxaban. Thus it is possible that once the decision was made to use rivaroxaban, several treatment related issues, such as scheduling outpatient INR checks, were no longer a potential barrier to discharge. This remains a potential confounder for this retrospective study.

Importantly, original power, which was defined as a total of 272 patients to achieve 80% power, was not reached due to lack of eligible rivaroxaban patients. However, the main risk associated with an underpowered study is the risk of a type II error which did not occur in this study because there was a significant difference in the primary outcome. Regardless, the results need to be interpreted with caution due to the low sample size, especially in subgroups.

The results of our study were consistent with those previously reported from post hoc analysis of the EINSTEIN-PE trial [14]. When specifically looking at the North American population, Bookhart and colleagues found a median one-day difference in LOS between those treated with rivaroxaban versus warfarin plus enoxaparin (3 versus 4 days) [14]. Our study is methodologically dissimilar, but the more pronounced LOS difference we observed may be better representative of real-world practice. The more inclusive criteria, such as allowing greater than 48 hours of enoxaparin prior to rivaroxaban initiation, likely increase the external validity of our results.

Lastly, there could be a large number of patients that were not screened for inclusion due to the fact that many patients did not receive an initial dose of rivaroxaban prior to discharge. Additionally, we likely excluded many stable patients who were discharged from the emergency department and avoided admission entirely. Patients who had a primary discharge diagnosis other than PE but were treated for acute PE during their hospitalization were also missed.

It is important to note that while rivaroxaban may decrease LOS, this may not be true of other NOACs, such as dabigatran or edoxaban, which require 5–10 days of parenteral anticoagulation prior to their initiation for the treatment of PE.

## 5. Conclusions

In conclusion, rivaroxaban use was associated with a decreased LOS compared to warfarin plus enoxaparin in the treatment of acute PE. This data supports the consideration of rivaroxaban use as a way to possibly shorten time to discharge.

## Figures and Tables

**Table 1:** Baseline characteristics.

Characteristic	Warfarin plus enoxaparin	Rivaroxaban
	(*N* = 82)	(*N* = 76)
Male [% (*N*)]	49 (40)	59 (45)
Age (years ± SD)	63 ± 15	55 ± 15
Classification [% (*N*)]		
Stable	74 (61)	79 (60)
Submassive	23 (19)	18 (14)
Massive	2 (2)	3 (2)
CrCl admission (mL/minute ± SD)	71 ± 24	80 ± 28
Hgb admission (gm/dL ± SD)	13 ± 2.0	13 ± 2.0
BMI (kg/m^2^ ± SD)	32 ± 9.0	32 ± 8.4
HFrEF [% (*N*)]	4 (3)	3 (2)
DM [% (*N*)]	23 (19)	11 (8)
HTN [% (*N*)]	63 (52)	49 (37)
CAD [% (*N*)]	15 (12)	3 (2)
History DVT/PE [% (*N*)]	23 (19)	20 (15)
INR discharge (±SD)	1.9 ± 0.9	N/A
INR ≥ 2 at discharge [% (*N*)]	44 (36)	N/A

DM: diabetes mellitus, BMI: body mass index, CAD: coronary artery disease, CKD: chronic kidney disease, CrCl: creatinine clearance, CVA: cerebrovascular accident, DVT: deep vein thrombosis, HFrEF: heart failure with reduced ejection fraction, Hgb: hemoglobin, HTN: hypertension, and PE: pulmonary embolism.

**Table 2:** Subgroup analysis of length of stay in patients with primary discharge diagnosis of PE.

Characteristic	Warfarin plus enoxaparin median LOS (days) (IQR)	Rivaroxaban median LOS (days) (IQR)	LOS *P* value
Age (years)			
≥60 (*N* = 51, 33)^*∗*^	4.5 (2.7, 5.9)	1.8 (1.2, 3.7)	<0.001
<60 (*N* = 31, 43)^*∗*^	4.0 (1.8, 5.9)	2.3 (1.6, 2.9)	<0.01
Classification			
Stable (*N* = 61, 60)^*∗*^	4.0 (2.6, 5.8)	1.8 (1.3, 2.8)	<0.001
Submassive (*N* = 19, 14)^*∗*^	5.0 (2.7, 7.7)	3.6 (1.8, 4.2)	0.058
Payer type			
Private (*N* = 25, 54)^*∗*^	3.7 (2.2, 5.8)	1.8 (1.5, 2.8)	0.002
Medicare/Medicaid (*N* = 40, 19)^*∗*^	4.1 (2.5, 6.1)	3.7 (1.8, 4.5)	0.093

^*∗*^ *N* = number in warfarin plus enoxaparin group and number in rivaroxaban group, respectively.

CrCl: creatinine clearance; IQR: interquartile range.

Massive PE not included due to low frequency of occurrences.

**Table 3:** Subgroup analysis of time interval from initial dose of oral anticoagulant to discharge in patients with primary discharge diagnosis of PE.

Characteristic	Warfarin plus enoxaparin median LOS (days) (IQR)	Rivaroxaban median LOS (days) (IQR)	Interval *P* value
Age (years)			
≥60 (*N* = 51, 33)^*∗*^	3.9 (2.7, 4.9)	0.9 (0.8, 2.1)	<0.001
<60 (*N* = 31, 43)^*∗*^	2.9 (1.6, 4.9)	0.8 (0.2, 1.1)	<0.001
Classification			
Stable (*N* = 61, 60)^*∗*^	3.0 (1.9, 4.8)	0.9 (0.5, 1.6)	<0.001
Submassive (*N* = 19, 14)^*∗*^	4.2 (3.0, 6.9)	0.9 (0.5, 2.2)	<0.001
Payer type			
Private (*N* = 25, 54)^*∗*^	2.9 (1.8, 4.9)	1.8 (1.5, 2.8)	<0.001
Medicare/Medicaid (*N* = 40, 19)^*∗*^	3.9 (1.9, 5.8)	3.7 (1.8, 4.5)	0.001

^*∗*^ *N* = number in warfarin plus enoxaparin group and number in rivaroxaban group, respectively.

CrCl: creatinine clearance; IQR: interquartile range.

Massive PE not included due to low frequency of occurrences.
